# Mechanisms of early glucose regulation disturbance after out-of-hospital cardiopulmonary resuscitation: An explorative prospective study

**DOI:** 10.1371/journal.pone.0214209

**Published:** 2019-03-25

**Authors:** Hanna Vihonen, Markku Kuisma, Ari Salo, Susanne Ångerman, Kirsi Pietiläinen, Jouni Nurmi

**Affiliations:** 1 Department of Emergency Medicine and Services, Päijät-Häme Central Hospital, Lahti, Finland; 2 Department of Emergency Medicine and Services, Helsinki University and Helsinki University Hospital, Helsinki, Finland; 3 Obesity Research Unit, University of Helsinki and Endocrinology, Abdominal Center, Helsinki University and Helsinki University Hospital, Helsinki, Finland; Azienda Ospedaliero Universitaria Careggi, ITALY

## Abstract

**Background:**

Hyperglycemia is common and associated with increased mortality after out-of-hospital cardiac arrest (OHCA) and return of spontaneous circulation (ROSC). Mechanisms behind ultra-acute hyperglycemia are not well known. We performed an explorative study to describe the changes in glucose metabolism mediators during the prehospital postresuscitation phase.

**Methods:**

We included patients who were successfully resuscitated from out-of-hospital cardiac arrest in two physician-staffed units. Insulin, glucagon, and glucagon-like peptide 1 (GLP-1) were measured in prehospital and hospital admission samples. Additionally, interleukin-6 (IL-6), cortisol, and HbA1c were measured at hospital admission.

**Results:**

Thirty patients participated in the study. Of those, 28 cases (71% without diabetes) had sufficient data for analysis. The median time interval between prehospital samples and hospital admission samples was 96 minutes (IQR 85–119). At the time of ROSC, the patients were hyperglycemic (11.2 mmol/l, IQR 8.8–15.7), with insulin and glucagon concentrations varying considerably, although mostly corresponding to fasting levels (10.1 mU/l, IQR 4.2–25.2 and 141 ng/l, IQR 105–240, respectively). GLP-1 increased 2- to 8-fold with elevation of IL-6. The median glucose change from prehospital to hospital admission was -2.2 mmol/l (IQR -3.6 to -0.2). No significant correlations between the change in plasma glucose levels and the changes in insulin (r = 0.30, p = 0.13), glucagon (r = 0.29, p = 0.17), or GLP-1 levels (r = 0.32, p = 0.15) or with IL-6 (r = (-0.07), p = 0.75), cortisol (r = 0.13, p = 0.52) or HbA1c levels (r = 0.34, p = 0.08) were observed. However, in patients who did not receive exogenous epinephrine during resuscitation, changes in blood glucose correlated with changes in insulin (r = 0.59, p = 0.04) and glucagon (r = 0.65, p = 0.05) levels, demonstrating that lowering glucose values was associated with a simultaneous lowering of insulin and glucagon levels.

**Conclusions:**

Hyperglycemia is common immediately after OHCA and cardiopulmonary resuscitation. No clear hormonal mechanisms were observed to be linked to changes in glucose levels during the postresuscitation phase in the whole cohort. However, in patients without exogenous epinephrine treatment, the correlations between glycemic and hormonal changes were more obvious. These results call for future studies examining the mechanisms of postresuscitation hyperglycemia and the metabolic effects of the global ischemic insult and medical treatment.

## Introduction

Hyperglycemia is common after out-of-hospital cardiac arrest (OHCA) and has been associated with a poor outcome [1]. An increasing trend in blood glucose before hospital admission is associated with mortality and highlights the significance of this ultra-acute phase of postresuscitation care [2]. OHCA and subsequent resuscitation causes metabolic derangements that have not previously been described in the ultra-acute phase.

Stress-induced hyperglycemia (SIH) is a well-established phenomenon that occurs during critical illness [3–5]. Hyperglycemia is associated with mortality in a U-shaped manner in critically ill patients with or without diabetes [1,3,6]. SIH is characterized by insulin resistance and an excess of the counterbalancing hormones of insulin, including glucagon, catecholamines, cortisol, and growth hormone [5,7]. Interleukin-6 (IL-6), along with tumor-necrosis factor-alpha (TNF-alpha), is thought to be an important cytokine in mediating the effect of SIH [5,8].

During OHCA and resuscitation, the patient is exposed to a global ischemia-reperfusion state that also affects the endocrine organs, such as the liver, pancreas and intestines, possibly leading to diminished hormonal production causing decreased endogenous insulin and GLP-1 levels. Giving exogenous epinephrine during resuscitation from OHCA may also affect the early phase of blood glucose disturbance. Thus, the mechanisms of hyperglycemia in the early postresuscitation phase may differ from those of SIH. Hence, this information could be necessary for designing possible interventions targeting ultra-acute hyperglycemia. The aim of this explorative study was to describe changes in glucose metabolism mediators during the prehospital phase after successful cardiopulmonary resuscitation from OHCA.

## Methods

### Study design

We conducted a prospective explorative study on biomarkers related to blood glucose metabolism in OHCA patients in the acute phase. The study protocol was approved by the Ethical Committee of Helsinki University Hospital (357/13/03/02/2012), and the study protocol was registered at clinicaltrials.gov (NCT01968148). Written informed consent was obtained from the next-of-kin on the scene. Due to the time-critical nature of the study design, in some cases, an informed verbal consent was asked from next-of-kin before blood sampling if written consent from next-of-kin was extremely impractical to obtain because of environmental factors. The consent was confirmed and signed as soon as it became practical during prehospital care. One additional member of the staff always witnessed the verbal consent. This practice is in line with the Finnish legislation on clinical studies. The studied biomarker samples were collected immediately after ROSC and at hospital admission. The relationship of the change in glucose levels with the studied biomarkers was then analyzed. The study protocol data can be reviewed at http://doi.org/10.5281/zenodo.2591961.

### Study setting and population

We included cardiopulmonary-resuscitated OHCA patients aged 18 years or older regardless of their initial rhythm or suspected cause. Study patients were recruited only on weekdays during office hours (from 8 a.m. to 4 p.m.), when necessary laboratory facilities for handling the study samples were available in the receiving hospitals. We excluded cases without next-of-kin on the scene to provide the consent to participate. The study protocol allowed the clinical staff to exclude a patient in a scenario where patient safety would have been compromised due to a lack of time to carry out the study procedures.

Helsinki University Hospital is responsible for organizing and supervising Emergency Medical Services (EMS) for the 1.6 million inhabitants of the Helsinki metropolitan area. EMS has two physician-staffed units. One is a helicopter emergency medicine service (HEMS) unit, and the other is a ground unit. Physician-staffed units are dispatched to all witnessed OHCA cases. This study was conducted in physician-staffed units.

### Key outcome measures

Prehospital blood samples were taken from the arterial line or by venous puncture immediately after ROSC and obtained consent. Blood samples for glucose and insulin analyses were drawn into citrate-fluoride and serum-gel tubes, respectively. Blood samples for glucagon and glucagon-like peptide 1 (GLP-1) analyses were drawn into PD P800 (Becton, Dickson and Company, NJ, USA) tubes, which contained esterase, protease and DPP-IV inhibitors for sample preservation until laboratory analysis. All tubes were covered in icepacks and transported to the emergency department to be delivered immediately to the central laboratory of the hospital. After the patients arrived at the hospital, samples for glucose insulin, glucagon, GLP-1, IL-6, cortisol, and HbA1c analyses were drawn from an arterial line or via venous puncture.

A routine central laboratory analysis was conducted for all prehospital samples except GLP-1. Plasma for GLP-1 analysis was separated promptly, frozen at -70 degrees celcius and stored in the laboratory for later analysis. GLP-1 analysis for prehospital and hospital admission samples was performed using an ELISA test (Human GLP-1 ELISA, BioVendor, Brno, Czech Republic) according to the manufacturer’s instructions. The laboratory reference values for the concentrations were as follows: insulin 10–20 mU/l, glucagon <209 ng/ml, GLP-1 1–2 ng/ml, and IL-6 <3.4 ng/l [9–11].

The exogenous epinephrine dose used during resuscitation was 1 mg intravenously according to European resuscitation guidelines [12]. The number of epinephrine doses was recorded by EMS personnel.

### Data analysis

Very few studies with a similar study design have been performed; therefore, to estimate an appropriate sample size for our study, we used the study design of Llompart-Pou JA et al. as a reference [13]. They recruited 60 patients, and as the expected survival rate was predicted to be 50%, we estimated that 40 cases needed to be recruited for our explorative study to describe changes in glucose metabolism mediators in the prehospital phase after successful cardiopulmonary resuscitation from OHCA. Due to slow recruitment, after the enrollment of 30 patients, we performed an unplanned interim analysis, and based on the high variability of the results of biomarker levels, the enrollment ceased as no significant changes were expected with original sample size.

**Fig 1:**
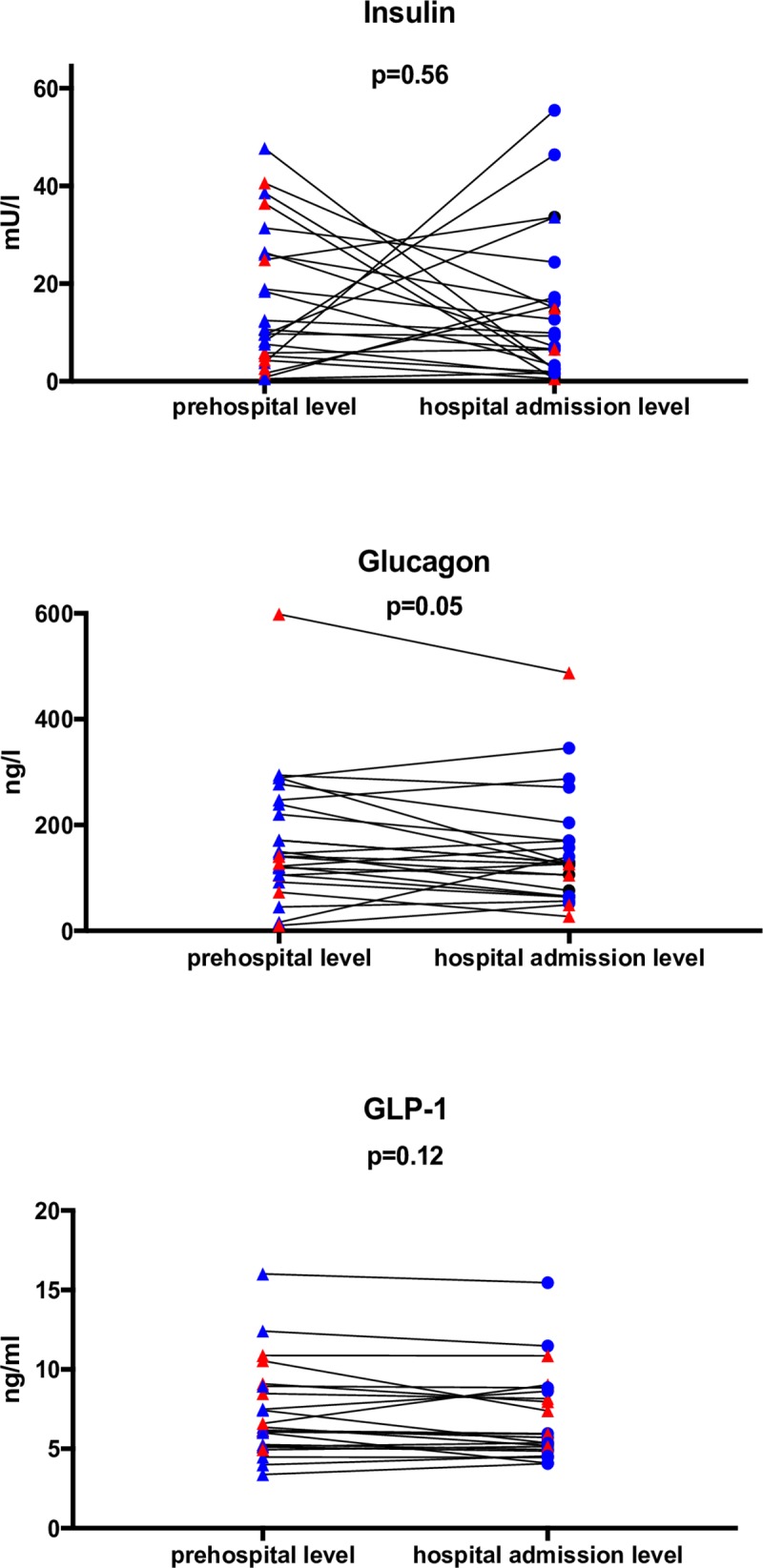
Prehospital and hospital admission levels of insulin, glucagon, GLP-1, and plasma glucose (N = 28). P-values were calculated by the Wilcoxon matched-pairs signed rank test.

Continuous variables are presented as medians and interquartile ranges (IQRs). Categorical data are presented as percentages with 95% confidence intervals, calculated with the modified Wald method. Continuous variables were compared by the Mann-Whitney test and Wilcoxon matched-pairs signed rank test (Fig 1 and Figure B in S1 File). Comparisons of categorical variables were performed with the chi-square test. Spearman’s correlation coefficients were calculated to analyze associations between continuous variables. The homeostasis model assessment (HOMA-IR) index was calculated to estimate insulin resistance = (insulin (mU/l) x glucose (mmol/l)/22.5) [14]. Two-tailed p-values <0.05 were considered statistically significant. All data were analyzed by GraphPad Prism 7.0.

## Results

### Demographics

During the study period from January 2014 to July 2015, we recruited 30 cardiopulmonary-resuscitated OHCA patients into the study. Two patients had missing laboratory data and were consequently excluded from the final analysis (Figure A in S1 File). Thus, the analyzed sample size was 28 patients, of whom, seven were known to have diabetes and one had newly diagnosed diabetes. Patients with diabetes had a median B-HbA1c level of 49 mmol/mol. All patients had type 2 diabetes. The characteristics of the patients are presented in Table 1. The median time interval between prehospital and hospital admission laboratory samples was 96 (IQR 85–119) minutes.

**Table 1:** Characteristics of the patients. Continuous variables are presented as medians and interquartile ranges (IQRs), and categorical variables are presented as percentages with 95% confidence intervals.

	All	Survivors	Nonsurvivors	p-value
**N**	28	17	11	
**Demographics**				
**Age, years**	71 (64–78)	71 (63–74)	72 (65–83)	0.334
**Gender, male (%)**	93 (76–100)	100 (78–100)	82 (51–96)	0.146
**BMI**	23.8 (20.9–29.7)	24.1 (20.9–30.7)	22.8 (20.7–24.8)	0.667
**Comorbidities**				
**Coronary disease**	36 (21–54)	41 (22–64)	27 (9–57)	0.689
**Hypertension**	57 (39–74)	41 (22–64)	82 (51–96)	0.054
**Heart failure**	11 (3–28)	18 (5–42)	27 (9–57)	0.002
**Chronic atrial fibrillation**	21 (10–40)	18 (5–42)	27 (9–57)	0.653
**OHCA**				
**Time to ROSC (minutes)**	20 (12–27)	17 (10–21)	27 (20–34)	0.004
**Initial rhythm VF**	82 (64–93)	94 (71–100)	64 (35–85)	0.062
**Interventions**				
**Mechanical ventilation**	64 (46–79)	53 (31–74)	82 (51–96)	0.226
**Induction of hypothermia**	14 (5–32)	12 (2–36)	18 (4–49)	1.000
**Vasoactive agents used**	79 (60–90)	65 (41–83)	100 (70–100)	0.055
**Number of epinephrine doses (1 mg each)**	1 (0–1)	0 (0–1)	3 (1–3)	0.001
**Bystander CPR**	50 (33–67)	65 (41–83)	27 (9–57)	0.120
**Last prehospital vital signs**				
**Systolic blood pressure (mmHg)**	122 (111–141)	124 (112–133)	119 (110–155)	0.936
**Diastolic RR (mmHg)**	70 (62–75)	70 (63–77)	65 (60–70)	0.258
**Heart rate (per minute)**	89 (66–105)	92 (67–105)	85 (63–107)	0.936
**Arterial oxygen saturation**	98 (95–100)	98 (95–100)	98 (95–100)	0.646

BMI = body mass index, OHCA = out-of-hospital cardiac arrest, ROSC = return of spontaneous circulation, CPR = cardiopulmonary resuscitation

### Change in blood glucose

All patients except two (prehospital plasma glucose levels of 2.6 mmol/l and 3.6 mmol/l) were initially hyperglycemic and remained hyperglycemic. Plasma glucose levels showed a decreasing trend in 22 patients and an increasing trend in 6 patients (Fig 1, Figure C in S1 File, and Figure D in S1 File).

### Hormone levels

The measured levels of the hormones and the changes that occurred during the prehospital phase are presented in Fig 1. No consistent and significant changes in the concentrations were observed despite large individual changes. As we hypothesized, the insulin concentration was close to the fasting level after ROSC, although insulin showed a marked change without any trend consistency among the patients. The HOMA-IR index revealed early insulin resistance during the prehospital phase in all cases. It presented in a U-shaped pattern, with increasing and decreasing levels at hospital admission.

**Fig 2:**
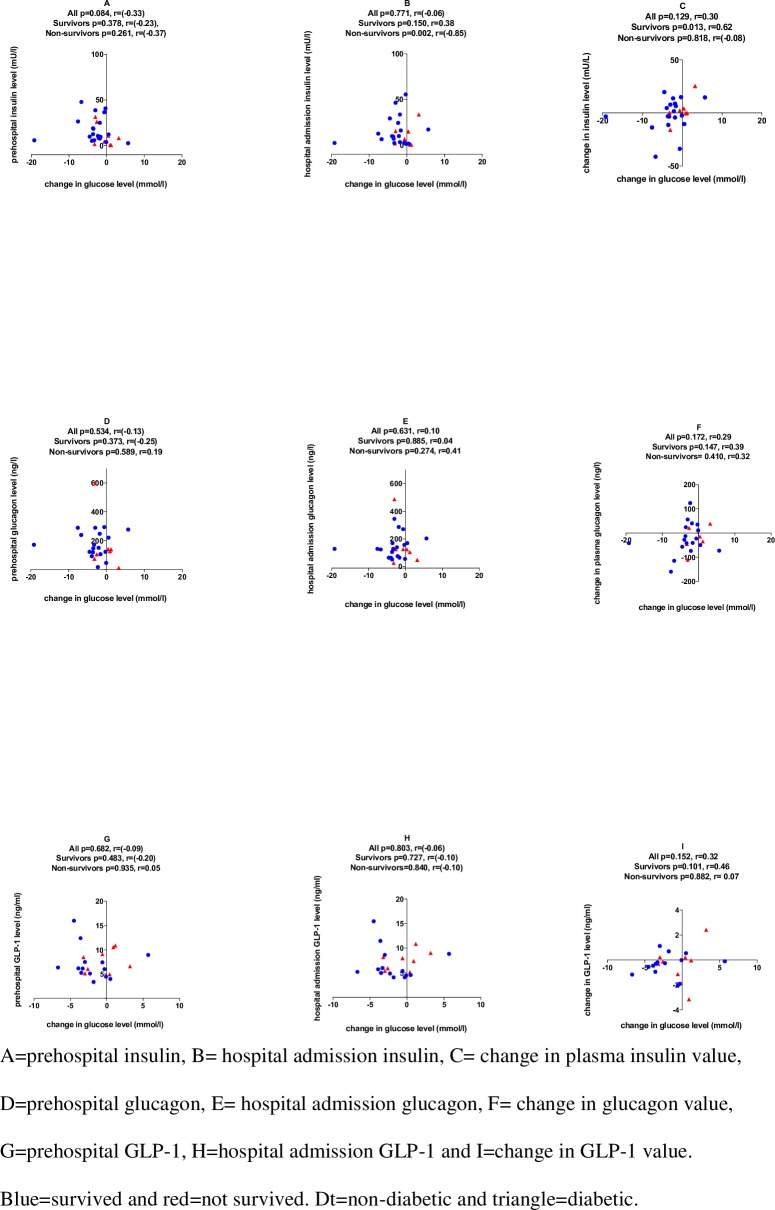
Correlation of insulin, glucagon and GLP-1 levels with the change in plasma glucose levels (N = 28).

**Fig 3:**
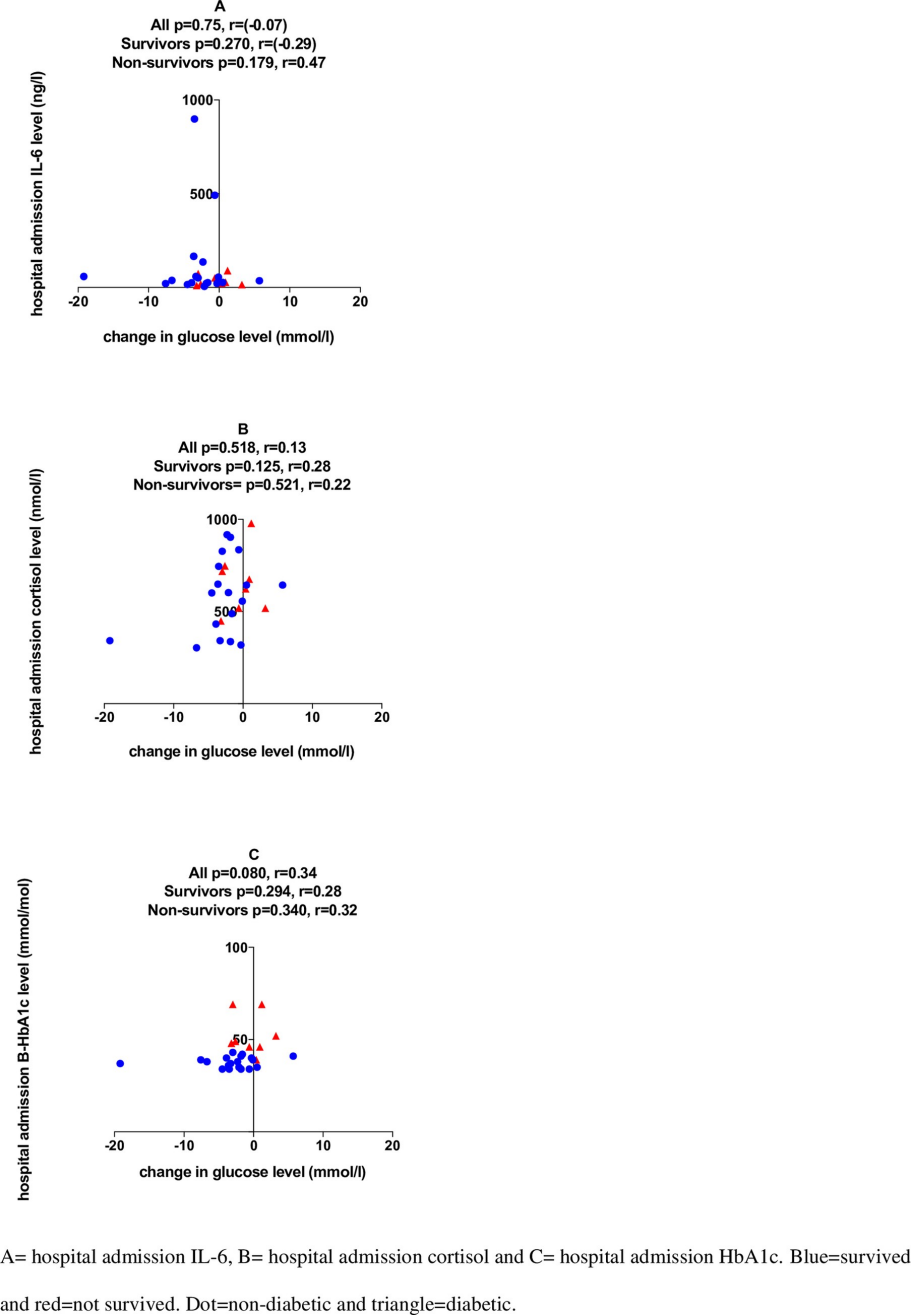
Correlation of IL-6, cortisol and B-HbA1c levels with the change in plasma glucose levels (N = 28).

The glucagon concentration was at a fasting level in the majority of patients. No significant correlations of the change in the glucose concentration with any of the measured hormone levels or with the change in the hormone levels was observed (Figs 2 and 3). However, prehospital GLP-1 levels were up to eight times higher than normal reference levels compared to normal healthy controls (6.3 ng/ml, IQR 5.2–9.0). Differences between the prehospital sample and the hospital sample showed that GLP-1 levels remained elevated with a nonsignificant median decrease of -0.2 (IQR -1 to 0.2) ng/ml. GLP-1 did not show any correlation with changes in blood glucose or other measured biomarkers.

IL-6 concentrations at hospital admission had high internal variation among the study sample (range: 6 to 899 ng/l). Overall, with few exceptions, IL-6 levels were consistently low, but above the postprandial level, with a median of 32 (IQR 22–63) ng/l (Table 2). Cortisol (range: 304 to 3475 nmol/l) and B-HbA1c (range: 34 to 69 mmol/mol) showed high internal variation among the cases as well. The levels of IL-6 and cortisol, markers of an inflammatory response, did not have a significant correlation with the change in the glucose concentration. Furthermore, as expected, the glucose concentration change was not correlated with the level of HbA1c, which is an indicator of chronic hyperglycemia (Fig 3). Except for cortisol, mortality did not affect significantly biomarker values (Table 2).

**Table 2:** Median values of the studied biomarkers from surviving and nonsurviving patients. Data are presented as the medians and interquartile ranges (IQRs).

Biomarkers	All	Survivors	Nonsurvivors	p-value
**N**	28	17	11	
**Insulin (mU/l)**				
**Prehospital**	10.2 (5.4–25.7)	9.6 (5.1–25.5)	12.2 (5.2–26.4)	0.917
**Hospital admission**	9.8 (2.4–24.4)	15.2 (3.4–31.3)	7.1 (1.7–12.7)	0.174
**Change**	0.3 ((-4.3)-8.7)	3.3 (-3.2–14.4)	-3.2 (-11.3–4.6)	0.119
**Glucagon (ng/l)**				
**Prehospital**	141 (106–243)	141 (73–239)	143 (121–289)	0.374
**Hospital admission**	127 (65–170)	126 (61–149)	129 (85–221)	0.466
**Change**	-32 ((-55)-23)	-41 (-73-35)	-28 (-54-6)	0.942
**GLP-1 (ng/ml)**				
**Prehospital**	6.3 (5.1–9.0)	6.1 (5.1–8.6)	7.5 (5.5–10.8)	0.238
**Hospital admission**	5.8 (4.9–8.7)	5.6 (4.8-.3)	6.6 (5.1–10.3)	0.482
**Change**	-0.2 ((-1.0)-0.2)	-0.2 (-1.0–0.2)	-0.4 (-1.7–0.40)	0.570
**IL-6 (ng/l)**				
**Hospital admission**	32 (22–63)	31 (20–58)	40 (24–248)	0.225
**Cortisol (nmol/l)**				
**Hospital admission**	623 (449–748)	519 (342–639)	745 (644–903)	0.004*
**HbA1c (mmol/mol)**				
**Hospital admission**	39 (36–46)	41 (38–48)	36 (34–43)	0.086

GLP-1 = glucagon-like-peptide-1, IL-6 = interleukin 6.

* represents a significant difference.

We additionally analyzed patients (n = 13) for whom exogenous epinephrine was not administered. In these patients, we found median hospital admission blood glucose levels of 8.1 (6.7–10.3) and 9.9 (8.3–12.2) in patients who received exogenous epinephrine, respectively. Exogenous epinephrine administration did not markedly affect HOMA-IR results, indicating a U-shaped pattern of insulin resistance; in half of the cases, insulin resistance increased, and in the other half of the cases, it decreased (Figure B and Figure C in S1 File). We also found that in this patient group, the change in blood glucose was correlated with the change in insulin (r = 0.59, p = 0.035) as well as with the change in glucagon (r = 0.65, p = 0.047), demonstrating that decreasing blood glucose values were associated with a simultaneous decrease in insulin and glucagon levels (Figure B and Figure C in S1 File). Changes in blood glucose did not correlate with IL-6, cortisol, or HbA1c when exogenous epinephrine was not administered (Figure D in S1 File).

## Discussion

The main finding of the current study was the lack of a single hormonal mechanism explaining the changes in blood glucose levels in the ultra-acute stage of the postresuscitation phase after OHCA. All measured hormone levels showed marked variability among the patients without correlation to the change in their blood glucose level, except when exogenous epinephrine was not administered during resuscitation for OHCA. In these cases, changes in blood glucose correlated with changes in insulin and glucagon.

Characteristics of SIH, including an increased inflammatory response with elevated IL-6, an excess of glucagon and other counterregulatory hormones and an initial relative insulin deficiency with the consequent development of insulin resistance in a form described in the previous literature, was only partly observed. Although, in some cases, relative insulin deficiency prior to developing insulin resistance seemed to be present, glucagon was not produced in excess. In contrast to our initial hypothesis of decreasing GLP-1 levels, GLP-1 increased along with IL-6 levels.

After OHCA and subsequent successful cardiopulmonary resuscitation, a condition called postcardiac arrest syndrome develops, and in the intensive care unit, SIH is encountered [15,16]. The degree of metabolic disturbance is a combination of two factors: the extent of ischemic injury and the quality of postresuscitation care [1]. Insulin resistance is thought to be a key contributor to SIH by providing glucose to vital organs during increased energy expenditure in periods of critical illness [17]. High levels of glucagon, cortisol, catecholamines and growth hormone preserve energy reserves and, hence, attempt to counterbalance the consequence of insulin resistance [5]. Complex inflammatory cascades are also present with SIH, and high IL-6 levels occur [5].

Elevated GLP-1 levels during critical illness have been reported by previous studies [10,11,18]. These studies reported a 1.4- to 8-fold increase in GLP-1 levels and a 2- to 10-fold increase in IL-6 levels within 30 minutes to 6 hours depending on the study design [10,11,18].

In contrast to previous findings, we found that insulin resistance, calculated by the HOMA-IR index, was present in half of the cases during the prehospital phase but decreased or was nonexistent at hospital admission. Independent of insulin resistance, the levels of insulin and glucagon were mainly at fasting levels. However, GLP-1 levels markedly increased along with increased secretion of IL-6. These results showed no correlation with the change in blood glucose levels, with the exception of cases that did not receive exogenous epinephrine.

As far as we know, this study was the first to observe metabolism in the ultra-acute phase after OHCA, whereas earlier observations were mainly from intensive care units, when the functionality of the endocrine organs may have recovered. We suggest four possible reasons why we observed differing blood glucose levels with high variability in the very early phase instead of common pathophysiological mechanisms of SIH. First, this may be a result of a global ischemic condition after OHCA, during which ischemia occurs in the endocrine organs, disturbing their normal function. Ischemic conditions cause oxidative stress, which injures mitochondria, instantly disturbing energy production [5]. Second, chronic prearrest comorbid conditions and flow/no flow time may have affected the homeostasis of the patients prior to OHCA for an unknown time period. Third, six patients received larger fluid volumes due to hypothermia induction during the prehospital phase, which may explain the early decrease in blood glucose levels in these individual cases but not in the rest of the cases [19]. Fourth, exogenous epinephrine seems to disturb the homeostasis of the body during the very early phases of blood glucose disturbance.

Active glucose control, most likely with mild permissive hyperglycemia (7 to 10 mmol/l), during critical illness leads to a better outcome than SIH or hypoglycemia in many patient groups. Hence, interventions to achieve mild hyperglycemia should be considered. By administering exogenous insulin, the altered insulin metabolism in SIH can be normalized, and it has been shown to benefit outcomes [20,21]. In time-critical illnesses, such as ischemic stroke, myocardial infarction and OHCA, the most effective time window for interventions may be the ultra-acute phase, i.e., the prehospital environment. We have demonstrated that even in urban and suburban areas with relatively short transport distances, blood glucose levels can be significantly affected by insulin infusion [20].

GLP-1 is an incretin hormone that causes insulin secretion and suppresses glucagon production in the body [22]. Previous studies have shown that endogenous GLP-1 levels increase during critical illness, preceded by an early rise of IL-6 levels, and this independently predicts mortality [10,11,18,23]. Increased endogenous GLP-1 seems to be beneficial. Perl et al. found that increased GLP-1 levels functioned as an adaptive inflammatory modulation response during the early stages of sepsis [10]. In contrast, the beneficial effect of endogenous GLP-1, higher endogenous GLP-1 levels have been associated with nonsurvivors compared to survivors [23]. However, this may be a result of decreased clearance during kidney failure [24]. Hence, GLP-1 seems to be a very important biomarker during the very early phase of blood glucose disturbance when coping with acute critical illness.

There have been promising studies using GLP-1 analogs for critical illness and OHCA instead of using insulin to treat SIH; GLP-1 analogs do not cause hypoglycemic episodes and carry possible beneficial antioxidative effects [25,26]. Wiberg et al. conducted a randomized controlled trial with 120 unconscious OHCA patients. They found that giving exenatide, a GLP-1 analog, was feasible. However, exenatide did not reduce mortality or neuronal damage [27]. The optimal intervention and timing of these GLP-1 analogs still require further research.

The main limitation of our study was the lack of a previous reference for a sufficient power calculation. For this reason, our sample size is potentially insufficient to elucidate the mechanisms behind glucose disturbance in the ultra-acute phase after OHCA. Due to the availability of laboratory facilities and consecutive slow enrollment, the sample size was limited to 30 cases after the interim analysis. Although the causes were likely multifactorial, we conducted a univariate analysis. This should be taken into consideration when interpreting the results. Other limitations were the inability to measure catecholamine levels, which most likely affect glucose metabolism after OHCA. A high number of confounding factors in glucose metabolism exist. Fasting levels of the hormones or medical conditions of the patients prior to OHCA were unknown, as was the prandial status of the patients. Antihyperglycemic medication, beta blockers, thiazide diuretics, atropine or corticosteroid medications may have affected the results [28], though it is highly unlikely that these factors would overcome the metabolic derangements of the glucose imbalance after OHCA.

Further studies should be conducted with a sufficient power calculation and a healthy control group. A multivariate analysis with a larger sample size should also be conducted to validate the results. In further studies, fasting levels, medical status, and other possible confounding factors should be taken into greater consideration. Finally, intervention studies on early glucose control after OHCA are required.

## Conclusions

Hyperglycemia was common after successful resuscitation from OHCA. Hormonal responses seemed to differ markedly among patients and seemed to lack specific hormonal mechanisms, except when exogenous epinephrine was not administered during resuscitation after OHCA. Overall, possibly due to global ischemic insult or unknown mechanisms, hyperglycemia in the early postresuscitation period seemed to differ from that commonly linked to SIH.

## Supporting information

S1 File (DOCX)
