# Supplementary material for: Mechanisms of early glucose regulation disturbance after out-of-hospital cardiopulmonary resuscitation: An explorative prospective study
Source: PLoS One. 2019 Mar 25;14(3):e0214209. doi: 10.1371/journal.pone.0214209 (PMC6433228; doi:10.1371/journal.pone.0214209)
Supplement: S1 File — (DOCX) [file pone.0214209.s001.docx]

**Figure A. Inclusion and exclusion of study patients.**

**Figure B. Prehospital-hospital admission values of insulin, glucagon, and GLP-1 without exogenously given epinephrine intravenous bolus. N=13.**

Blue=survived and red=not survived. Dot=non-diabetic and triangle=diabetic.

**Figure C. Correlation of insulin, glucagon and GLP-1 to change of glucose value without exogenously given intravenous bolus of epinephrine. All N=13 and survivors n=11. * marked as significant correlation.**

A=prehospital insulin, B=hospital admission insulin, C=change in insulin value, D=prehospital glucagon, E=hospital admission glucagon, F=change in glucagon value, G=prehospital GLP-1, H=hospital admission GLP-1 and I=change in GLP-1 value. Blue=survived and red=not survived. Dot=non-diabetic and triangle=diabetic.

**Figure D. Correlation of IL-6, cortisol and B-HbA1c to change of glucose value without exogenously given intravenous bolus of epinephrine. All N=13 and survivors n=11.**

A=insulin, B=glucagon and C=GLP-1. Blue=survived and red=not survived. Dot=non-diabetic and triangle=diabetic.
